# Studies of parenchymal texture added to mammographic breast density and risk of breast cancer: a systematic review of the methods used in the literature

**DOI:** 10.1186/s13058-022-01600-5

**Published:** 2022-12-30

**Authors:** Akila Anandarajah, Yongzhen Chen, Graham A. Colditz, Angela Hardi, Carolyn Stoll, Shu Jiang

**Affiliations:** 1 Division of Public Health Sciences, Department of Surgery, Washington University School of Medicine, 660 S Euclid Ave MSC 8100-0094-2200, Saint Louis, MO 63110 USA; 2 Saint Louis University School of Medicine, Saint Louis, MO USA; 3 Bernard Becker Medical Library, Washington University School of Medicine, MSC 8132-12-01, 660 S Euclid Ave, Saint Louis, MO 63110 USA

**Keywords:** Breast density, Mammography, Parenchymal patterns, Risk prediction, Texture

## Abstract

This systematic review aimed to assess the methods used to classify mammographic breast parenchymal features in relation to the prediction of future breast cancer. The databases including Medline (Ovid) 1946-, Embase.com 1947-, CINAHL Plus 1937-, Scopus 1823-, Cochrane Library (including CENTRAL), and Clinicaltrials.gov were searched through October 2021 to extract published articles in English describing the relationship of parenchymal texture features with the risk of breast cancer. Twenty-eight articles published since 2016 were included in the final review. The identification of parenchymal texture features varied from using a predefined list to machine-driven identification. A reduction in the number of features chosen for subsequent analysis in relation to cancer incidence then varied across statistical approaches and machine learning methods. The variation in approach and number of features identified for inclusion in analysis precluded generating a quantitative summary or meta-analysis of the value of these features to improve predicting risk of future breast cancers. This updated overview of the state of the art revealed research gaps; based on these, we provide recommendations for future studies using parenchymal features for mammogram images to make use of accumulating image data, and external validation of prediction models that extend to 5 and 10 years to guide clinical risk management. Following these recommendations could enhance the applicability of models, helping improve risk classification and risk prediction for women to tailor screening and prevention strategies to the level of risk.

**Supplementary Information:**

The online version contains supplementary material available at 10.1186/s13058-022-01600-5.

## Introduction

Evolving technology from film mammograms to digital images has changed the sources of data and ease of access to study a range of summary measures from breast mammograms and the risk of breast cancer [1, 2]. These include more extreme measures of density and also measures of breast texture features. In particular, as women have repeated mammograms as part of a regular screening program [3–5], access to repeated images including changing texture features has become more feasible in real time for risk estimation and classification and appropriately counseling women for their risk management [6–8]. Using these features may facilitate improvement in risk classification [9] and hence more fully support precision prevention for breast cancer [8, 10].

The leading measure for long-term risk categorization extracted from mammograms is breast density [11, 12]. This is now widely used and reported with many states requiring the return of mammographic breast density measures to women as part of routine screening [13]. Mammographic breast density is a strong reproducible risk factor for breast cancer across different approaches used to measure it (clinical judgment or semi/automated estimation) [11] and across regions of the world [11, 14, 15]. However, density is known to be affected by confounders such as age and body mass index [16, 17]. Texture features within mammograms have been much less frequently studied for their contribution to risk stratification and risk prediction but could potentially be less impacted by these confounders. Moreover, mammogram density only aims to measure the relative amount of fibroglandular tissue in the breast [18], which limits the ability to fully capture heterogeneity between patients in the breast tissue, while patterns of breast parenchymal complexity, formed by the x-ray attenuation of fatty, fibroglandular, and stromal tissues, are known to be associated with breast cancer [19, 20].

In 2016, Gastounioti and colleagues summarized the literature at that time to classify approaches to parenchymal texture classification: (1) gray-level features—skewness; kurtosis; entropy; and sum intensity, (2) co-occurrence features—entropy; inertia; difference moment; and coarseness, (3) run-length measures gray-level non-uniformity and run-length non-uniformity, (4) structural patterns measures lacunarity, fractal dimension, and (5) multiresolution spectral features [21]. They conclude from this review that multiparametric texture features may be more effective in predicting breast cancer than single-group features. Although this review included studies using the ipsilateral and/or contralateral breast, it did not report on their time horizon. To address this and use of more comprehensive summaries of these features since their review in 2016, we conducted a systematic review of published studies.

In current medical practice, risk prediction is viewed as an objective way to assess the risk of a patient developing a disease such as a 10-year risk of cardiovascular disease [22]. Risk prediction models are often evaluated in terms of calibrations and discrimination. For instance, the AUC (a discrimination measure) describes how well a given model separates events from non-events. On the other hand, association measures such as a significant hazard ratio for an individual risk factor do not necessarily translate to a significant increase in discrimination performance when it is added to a prediction model [23].

We aim to summarize the methods used to classify mammographic breast parenchymal features in relation to the prediction of future breast cancer, the time from mammogram to the diagnosis of breast cancer, the analysis of data from either one or both breasts (averaged or assessed individually), the study design, and the statistical methods for estimating the association between features and risk. We then identify gaps in evidence to prioritize future studies and speed us to better support the precision prevention of breast cancer.

## Methods

### Eligibility criteria

#### Population

We considered all studies of adult women (at least 18 years old) involving original data. Abstract-only papers, review articles, and conference papers were excluded.

#### Intervention

We included studies measuring at least one non-density mammographic feature. A study had to explicitly define the mammographic features included. Studies that did not do this, such as those which used a deep model, were excluded because we would be unable to determine the characteristics of features used and compare these to other studies.

#### Comparison

We compared models using parenchymal texture features to those that did not.

#### Outcomes

Our primary outcome of interest was the risk of breast cancer, including both invasive and in situ cancers. The risk of breast cancer was required to be dichotomized (yes/no). Analysis of other risks (e.g., risk of interval vs. screen-detected cancer) and studies examining the association between mammographic features and other risk factors were excluded to narrow the scope of our paper.

Only studies available in English were included. Additionally, only studies published from 2016 onward were included to avoid overlap with previous reviews.

### Information sources

The published literature was searched using strategies designed by a medical librarian for the concepts of breast density, mammography, and related synonyms. These strategies were created using a combination of controlled vocabulary terms and keywords, and were executed in Medline (Ovid) 1946-, Embase.com 1947-, CINAHL Plus 1937-, Scopus 1823-, Cochrane Library (including CENTRAL), and Clinicaltrials.gov. Results were limited to English using database-supplied filters. Letters, comments, notes, and editorials were also excluded from the results using publication type filters and limits.

### Search strategy

An example search is provided below (for Embase).

('breast density'/exp OR ((breast NEAR/3 densit*):ti,ab,kw OR (mammary NEAR/3 densit*):ti,ab,kw OR (mammographic NEAR/3 densit*):ti,ab,kw)) AND ('mammography'/deOR mammograph*:ti,ab,kwOR mammogram*:ti,ab,kwOR mastrography:ti,ab,kwOR ‘digital breast tomosynthesis’:ti,ab,kwOR ‘x-ray breast tomosynthesis’:ti,ab,kw)NOT('editorial'/it OR 'letter'/it OR 'note'/it) AND [english]/lim

The search was completed for the first time on September 9, 2020, and was run again on October 14, 2021, to retrieve citations that were published since the original search. The second search was dated limited to 2020-present (October 2021). Full search strategies are provided in the Additional file 1: appendix.

### Selection process

Two reviewers (AA, CS) worked independently to review the titles and abstracts of the records. Next, the two reviewers independently screened the full text of the articles that they did not reject to determine which were eligible for inclusion. Any disagreements about which articles to include were resolved by consensus. Two reviewers (AA, YC) then went through this subset independently and excluded the ones without explicitly defined features.

### Data collection process

We created a data extraction sheet that two reviewers (AA, YC) used to independently extract data from the included studies. Disagreements were resolved by a third reviewer. If included studies were missing any desired information, any additional papers from the works cited, such as previous reports, methods papers, or protocols, were reviewed for this information.

### Data items

Any estimate of the risk of breast cancer was eligible to be included. Risk models could combine multiple texture features or examine texture features individually. The predictive ability could be evaluated using an area under the curve, odds ratio, matched concordance index, hazard ratio, or *p* value. No restrictions on follow-up time were placed. For studies that reported multiple risk estimates, we prioritized the area under the curve with the most non-mammogram covariates included from the validation study if applicable. If the study did not report an area under the curve, we listed the primary models which were discussed in the Results section of the paper.

We collected data on:

- the report: author, publication year
- the study: location/institution, number of cases, number of controls, study design
- the research design and features: lapsed time from mammogram to diagnosis
- the mammogram: machine type, mammogram view(s), breast(s) used for analysis
- the model: how density was measured, number of texture features extracted, types of texture features extracted, whether feature extraction was machine or human, whether all features were used in the analysis, how features for analysis were chosen, non-mammogram covariates included, established confounders for density, prediction horizon, statistical methods for assessing risk association

### Risk of bias

The quality of the included studies was assessed using the Quality in Prognostic Studies (QUIPS) tool [24]. Risk of bias was rated as high, moderate, low, or unclear by two reviewers (AA and CS) across six domains including study participation, study attrition, prognostic factor measurement, outcome measurement, study confounding, and statistical analysis and reporting. Raters independently recorded supporting information and justification for judgments of risk of bias for each domain. Any disagreements were resolved by consensus.

### Human subjects

This study did not involve human subjects, and therefore oversight from an Institutional Review Board was not required.

### Registration and protocol

This review was not registered and a protocol was not prepared.

## Results

**Fig. 1:**
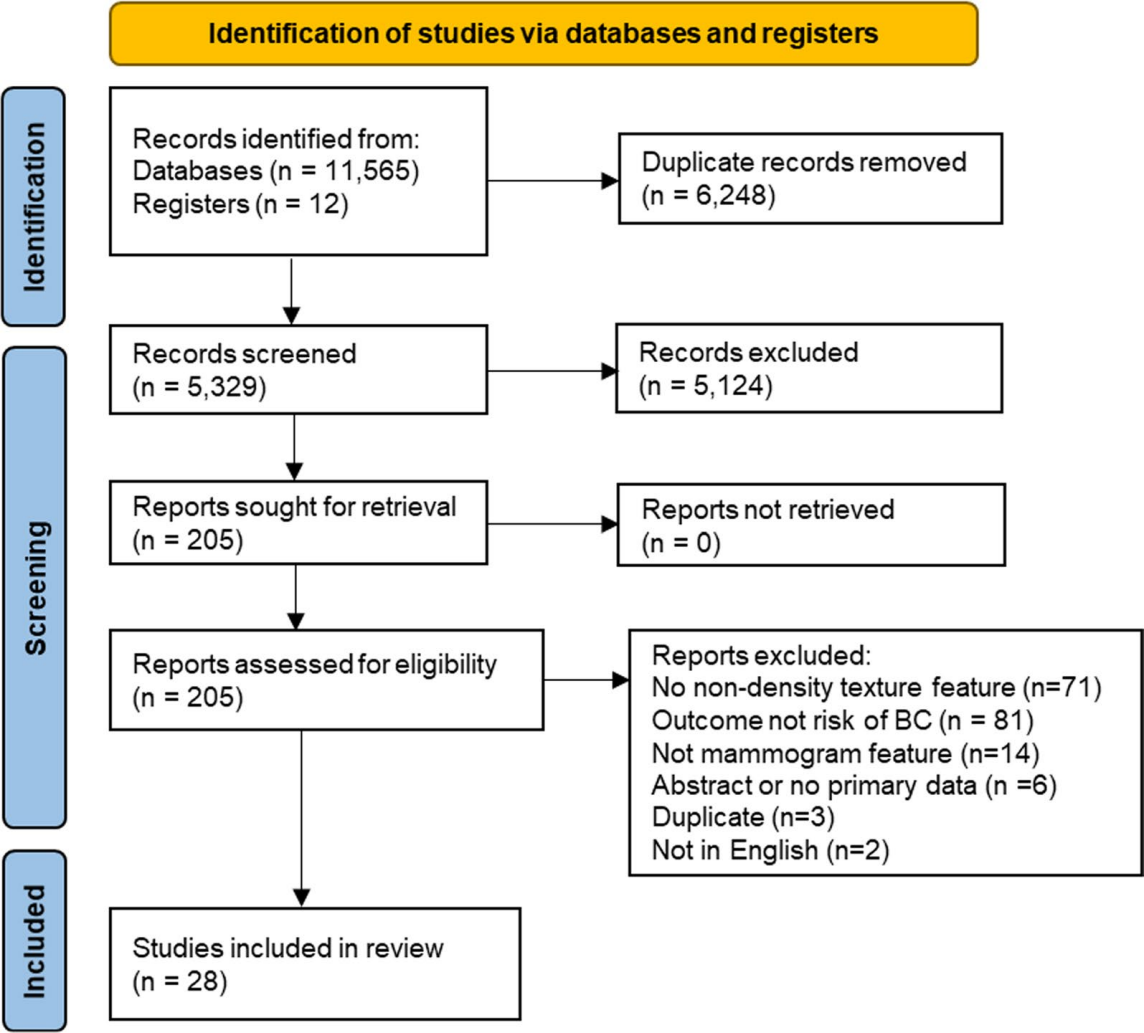
PRISMA 2020 Flow Diagram for identification of included studies

The search and study selection process is shown in Fig. 1. A total of 11,111 results were retrieved from the initial database literature search and imported into Endnote. Eleven citations from ClinicalTrials.gov were retrieved and added to an Excel file library. After removing duplicates, 4863 unique citations remained for analysis. The search was run again in October 2021 to retrieve citations that were published since the original search. A total of 1633 results were retrieved and imported to Endnote. After removing duplicates, including duplicates from the original search, 466 unique citations were added to the pool of results for screening. Between the two searches, a total of 11,577 results were retrieved, and there were 5329 unique citations.

Of the 5329 unique citations, 5124 were excluded based on the review of the title and abstract. Two hundred and five full-text reports were retrieved and assessed for eligibility by two readers. Of these, 177 were excluded for reasons such as not measuring a non-density feature, not reporting risk of breast cancer as an outcome, being an abstract or a duplicate paper, or not being published in English.

We identified 28 studies published since 2016 that met eligibility criteria as set out in the selection flowchart [25–52].

Of the 28 studies, only 7 were based on digitized analog film images [26, 38, 40, 47–49], 2 did not provide details [34, 37], and the others used full-field digital mammograms from Hologic, GE, or other manufacturers, or did not report details (see Table 1). The number of cases included in studies ranged from 20 up to 1900. Of the 28 studies, 8 included fewer than 100 cases [30, 35, 37, 41, 42, 46, 50, 51]. Most studies used a case–control design, although there were a few prospective and retrospective cohort studies.

**Table 1:** Twenty-eight studies of breast texture features classified from mammograms included in systematic review (sorted by year published)

References	Year	City/institution	Study design	Machine type	View used (CC/MLO/both)	# cases	# controls
Choi et al. [28]	2016	University of Ulsan	Retrospective cohort	General Electric Senographe DS and film	Both	240	0
Malkov et al. [38]	2016	USA	Case–control studies with 2 of 5 nested in cohorts	Film	CC	1171	1659
Tan et al. [43]	2016	University of Pittsburgh Medical Center	Case–control	Digital (not specified further)	Both	159	176
Winkel et al. [49]	2016	Bispebjerg Hospital	Prospective cohort with case–control sampling	Film	Both	121	259
Ali et al. [26]	2017	Sweden	Main analysis: case–control. Validation study: prospective cohort	Film for main analysis, GE Senographe Essential for validation study	MLO	1170 for main analysis, 69 for validation study	1283 for main analysis, 231 for validation study
Eriksson et al. [29]	2017	Sweden	Prospective cohort with case–control sampling	Digital (not specified further)	Both	433 for model development. An additional 137 women lacking information were included in calculating the absolute risk estimates	1732 when comparing study participant characteristics and mammographic features, 60,237 for calculating the absolute risk estimate
Wang et al. [45]	2017	National Health Service	Prospective cohort with case–control sampling	GE Senographe system	CC	264 for training case–control study, 317 for validation case–control study	787 for training, 931 for validation
Winkel et al. [48]	2017	Copenhagen, Denmark	Prospective cohort of false positives with case–control sampling	Film	Both	288	288
Yan et al. [50]	2017 (August)	NR	Case–control	Hologic Selenia	CC	83	85
Yan et al. [51]	2017 (October)	NR	Case–control	Hologic Selenia	CC	83	85
Gastounioti et al. [31]	2018	University of Pennsylvania	Case–control	Hologic Selenia Dimensions	MLO	115	460
Heidari et al. [32]	2018	NR	Case–control	Digital (not specified further)	CC	250	250
Li et al. [37]	2018	Fudan University Shanghai Cancer Center	Nested case–control prospective cohort	NR	CC	84	987
Schmidt et al. [40]	2018	Australia and Hawaii, USA	Case–control and nested case–control in prospective cohort	Film	CC	1236	2931
Tagliafico et al. [42]*	2018	Italy	Case–control	Hologic Selenia Dimensions	NR	20	20
Ward et al. [46]	2018	National Health Service	Prospective cohort	Hologic Selenia	Both	34	746
Evans et al. [30]	2019	Bradford (UK) Teaching Hospitals NHS Foundation Trust	Case–control	Digital (not specified further)	Mix of CC and MLO	58 images from 35 patients with cancer. Experiment 1D included an additional 50 abnormal mammograms with visible cancerous lesions taken from 50 patients	58 images from 35 patients without cancer. Experiment 1D included an additional 50 normal mammograms taken from 50 patients
Hsu et al. [34]	2019	University of California, Los Angeles	Prospective cohort	NR	NR	463 biopsy results	1675 biopsy results
Kontos et al. [35]	2019	University of Pennsylvania for case–control sample for evaluating associations to breast cancer, NR for screening sample for phenotype identification	Sample used in evaluating associations to breast cancer: case–control. Sample used for phenotype identification: cross-sectional	Hologic Selenia Dimensions for screening sample, GE Senographe 2000D and DS for case–control sample	Both for screening sample, NR for case–control sample	Screening sample included 18 detected cases with 12 in the training sample and 6 in the testing sample. 76 cases were in case–control sample	Screening sample included 2011 controls with 1327 in the training sample and 684 in the testing sample. 158 controls were in case–control sample
Perez-Benito [39]	2019	Valencian Community, Spain	Case–control from population screening program	FUJIFILM, IMS s.r.l., Giotto IRE, HOLOGIC, SIEMENS, or unknown	Both	808 cases with 606 in training set and 202 in test set	755 with 566 in training set and 189 in test set
Pertuz et al. [52, 63]	2019	Tampere University Hospital	Case–control	Philips MicroDose SI or General Electric Senographe Essential	CC	114	114
Tan et al. [44]	2019	Subang Jaya Medical Center	Case–control	Hologic Selenia	CC	250	250
Abdolell et al. [25]	2020	NR	Case–control from population screening program	Siemens MAMMOMAT Inspiration or MAMMOMAT Novation DRimaging system	Both	1882	5888
Ma et al. [36]	2020	Nanfang Hospital	Case–control	Hologic Selenia Dimensions	Both	608 for risk model development, 203 for validation	1261 for risk model development, 421 for validation
Sorin et al. [41]	2020	NR	Retrospective cohort	GE Senographe Essential	Both	53	463
Azam et al. [27]	2021	Sweden	Prospective cohort	General Electric, Philips, Spectrum Hologic, and Siemens	Both	676	52,597
Heine et al. [33]	2021	Mayo Clinic for first case–control study, the San Francisco Mammography Registry for generalization study	Case–control	Hologic Selenia	CC	514 for first study, 1474 for generalization study	1377 for first study, 2942 for generalization study
Warner et al. [47]	2021	USA	Prospective cohort with case–control sampling	Film	CC	1900	3921

*Tagliafico 2018 used digital breast tomosynthesis

*CC* craniocaudal, *MLO* mediolateral oblique, *NR* not reported

Tables 2 and 3 summarize how the texture features were identified in the mammographic images in studies not using the contralateral breast and those which did, respectively. These methods included defined masses or calcifications [29], or a predefined list of texture features such as 34 [31] or 44 [32] or even 112 [45] or 944 [44] initial features. Machine-driven identification of features was also reported. After machine identification, the features were reduced for analysis often based on statistical rules.

**Table 2:** Features of mammographic breast images used to assess breast texture features in addition to mammographic breast density and breast cancer risk for 23 studies not using the contralateral breast (sorted by year)

References	Time from mammogram to cancer diagnosis	Side used	Density (BIRAD categories/continuous)	Number of texture features extracted (other than density)	Types of texture features extracted (list all)	Machine or human extraction	All texture features used in the model (yes/no)	How features for analysis are chosen
Choi et al. [28]	Mean = 9.7 months (range 6–15 months)	NR	BIRADS	8	Normal appearing tissue, benign-appearing calcification, mass, calcification, architecture distortion, focal asymmetry	Human	Yes	N/A
Malkov et al. [38]	Mean = 5.1 years	Avg	Cumulus and custom software comparable to Cumulus	46	First- and second-order features, Fourier transform, and fractal dimension analysis	Machine	No	AUCs for each feature individually given
Tan et al. [43]	The average elapsed time between the “current” and each of “prior” #1, #2 and #3 studies was 1.16 ± 0.41, 2.30 ± 0.55 and 3.44 ± 0.72 years, respectively	Both	Computer-aided detection scheme	158	Mammographic density, structural similarity, and texture based image features	Machine	No	Stepwise regression analysis
Winkel et al. [49]	Average = 26 months (range 4–45 months)	Both	BIRADS	1	Tabár classification of parenchymal patterns	Human	Yes	N/A
Eriksson et al. [29]	Median = 1.74 years	Both	BIRADS and STRATUS	2	Calcifications and masses	Machine	Yes	N/A
Winkel et al. [48]	Average = 82.0 months, median = 75.5 months, range = 5 to 192 months	Both	BIRADS and Cumulus-like approach	1	Tabár classification of parenchymal patterns	Human	Yes	N/A
Yan et al. [50]	The interval between the prior (negative) and current (cancer detected) examinations are 410.0 ± 51.7 days for cases	Both	BIRADS	148	Bilateral mammographic tissue asymmetry maximum features	Machine	No	WEKA data mining and machine learning software package
Yan et al. [51]	12–36 months	Both	Mutual threshold	220	Asymmetry, mean and maximum features	Machine	No	WEKA data mining and machine learning software package
Gastounioti et al. [31]	Average = 1.9 years ± 0.7. Cases had negative screening mammograms at least one year prior to their diagnosis	Avg	BIRADS, LIBRA, and Quantus	34	Anatomically oriented texture features	Machine	No	Identified pairs of features with absolute Pearson correlation greater than 0.90 and for each pair removed the feature with the lowest variability in terms of its interquartile range. Starting from the remaining features, elastic net regression with nested cross-validation was used to build a parsimonious logistic regression model with the most discriminatory subset of covariates
Heidari et al. [32]	The time interval between the “prior” and “current” mammography screenings ranged from 12 to 18 months	Both	BIRADS	44	Bilateral asymmetry of mammographic tissue density distribution	Machine	No	Locally preserving projection based feature combination algorithm
Li et al. [37]	NR	Both	AutoDensity	1	Breast area	Machine	Yes	N/A
Schmidt et al. [40]	Melbourne: Cases were diagnosed, on average, 8 years after baseline interview (range, 3 months–16 years), and mammography was performed, on average, 2.8 years (standard deviation, 2.6 years; range, 0–14 years) after baseline. Australia: average 4 years for 32% of cases, and for the other affected women we used the mammogram from the opposite side to that in which the cancer was diagnosed. Hawaii: The mean time between the earliest mammogram and the breast cancer diagnosis was 6.3 years, while the earliest and the latest mammogram were, on average, 5.1 years apart for cases	Avg	Cumulus	20	Gray-level co-occurrence matrix textural features	Machine	Yes	N/A
Tagliafico et al. [42]	Cancer was detected at tomosynthesis	Avg	BIRADS	104	Radiomics features including skewness, energy, entropy, kurtosis, 90 percentile and dissimilarity	Machine	No	Selected to reduce the risk of over-fitting and according to features previously used to associate breast parenchymal patterns with cancer risk
Ward et al. [46]	NR	Both	Volpara	1	Patterns of parenchymal tissue	Human	Yes	N/A
Evans et al. [30]	Mammograms used were acquired 3 years prior to the mammograms that had revealed visible and actionable cancer	Both	Scale similar to BIRADS used	1	Non-localizable global gist signal	Human	Yes	N/A
Hsu et al. [34]	A false-positive biopsy recommendation was defined by the lack of cancer within 1 year of the screening examination	NR	BIRADS	5	Presence of lump, mass, calcification, architecture distortion, asymmetry	Human	No	Presence of lump included in final model. Mass, calcifications, architecture distortion, asymmetry examined individually with PPV values given
Kontos et al. [35]	For screening sample, within 1 year. Not specified for case–control sample	Avg	BIRADS and LIBRA	29	Phenotypes of mammographic parenchymal complexity based on four main types of features: histogram, co-occurrence, run-length, and structural	Machine	No	Excluded features with extremely low variation and those with extreme skewness
Abdolell et al. [25]	NR	NR	Densitas	1	Breast volume	Machine	Yes	N/A
Ma et al. [36]	At least 1 year later for validation	Both	BIRADS	1	Normalized average glandular dose	Machine	Yes	N/A
Sorin et al. [41]	Cancer cases were defined as all cancers detected at the time of contrast-enhanced spectral mammography imaging as well as cancers diagnosed during the follow-up period. Controls had at least 1-year follow-up	Both	BIRADS	1	Background parenchymal enhancement	Human	Yes	N/A
Azam et al. [27]	The median number of years between the last negative mammogram and the date of diagnosis was 2.8	Both	STRATUS	1	Microcalcification clusters	Machine	Yes	N/A
Heine et al. [33]	At least 6 months	Avg	Volpara	4	Variation measures	Machine	No	The two variants of V produced similar findings so only one was discussed in the results
Warner et al. [47]	Median = 4.1 years	Both	Cumulus and automated computer algorithm	1	Gray-scale variation	Machine	Yes	N/A

*AUC* area under the curve, *average* avg, *BIRADS* Breast Imaging Reporting and Data System, *LIBRA* Laboratory for Individualized Breast Radiodensity Assessment, *N/A* not applicable, *NR* not reported, *PD* percent density, *PPV* positive predictive value

**Table 3:** Features of mammographic breast images used to assess breast texture features in addition to mammographic breast density and breast cancer risk for 5 studies using the contralateral breast (sorted by year)

References	Time from mammogram to cancer diagnosis	Side used	Density (BIRAD categories/continuous)	Number of texture features extracted (other than density)	Types of texture features extracted (list all)	Machine or human extraction	All texture features used in the model (yes/no)	How features for analysis are chosen
Ali et al. [26]	Less than 3 years before diagnosis (and at latest, at date of diagnosis)	Contralateral for cases, random side chosen for controls	Cumulus and automated measure of area PD	13	Spatial organization of dense vs. fatty regions of the breast	Machine	Yes for AUC given, no for further analysis	Stepwise selection procedure
Wang et al. [45]	Training study: diagnosed at the same time as mammogram. Validation study: average = 3.0 years	Training: contralateral for cases and the left for controls. Validation: contralateral for cases and the same side for controls	Volpara	112	Features based on a gray-level co-occurrence matrix, neighborhood gray-tone difference matrix, form and shape of breast boundary, run-length, and gray-level size zone matrix, and statistical moments of pixel values	Machine	No	Selected from training set using least absolute shrinkage and selection operator
Perez-Benito [39]	NR	Contralateral	DMScan	23	Geometrical features and a global feature based on local histograms of oriented gradients	Machine	Yes	N/A
Pertuz et al. [52, 63]	NR	Contralateral for cases, right for controls	Cumulus-like approach	37	Parenchymal features including computational features and imaging parameters related to the mammographic system (compressed breast thickness, compression force, X-ray tube voltage peak and target–filter combination)	Machine	Yes	N/A
Tan et al. [44]	Within a year	Contralateral	Volpara	944	Gray-level co-occurrence matrix features, structural/pattern measures, gray-level intensity/histogram features, run-length features, and multiresolution/spectral features	Machine	No	Stepwise regression analysis

*AUC* area under the curve, *N/A* not applicable, *PD* percent density

The approach to the assessment of breast parenchymal texture and side of body (ipsilateral or contralateral breast) varied across studies. Almost half of the studies (13 out of 28) used BIRADs as the baseline measure of density [28–32, 34–36, 41, 42, 48–50]. Others used Volpara and machine-derived density or Cumulus-like approaches. Time from mammogram to diagnosis of breast cancer ranged from under 24 months on average up to 82 months.

We next assessed the value added from the addition of texture features to prediction models for breast cancer. As noted in Tables 2 and 3, there was a substantial variation in the number of texture features included and the method of their identification for inclusion (human-defined or machine-identified). Many papers only reported on the association of texture with the risk of breast cancer using an odds ratio or relative risk. These were often contemporaneous with diagnosis (measured on contralateral breast) [26, 39, 44, 45, 52]. Model building details and results were not routinely reported. Tables 4 and 5 give the AUC for a baseline model without texture and then the value for the model with texture added when these were reported separately. Studies were not comparable across time horizon and baseline models. Hence, we did not proceed to a numerical quantitative summary such as meta-analysis of AUC values. However, within studies we saw that those that reported concordance statistics for models primarily reported results of models with MD and then MD plus texture features. In these studies, we saw an increase in reported concordance when texture features were added. The addition of parenchymal features often increased the AUC by 0.05 in studies not using the contralateral breast as presented in Table 6. Several studies using the contralateral breast at the time of cancer diagnosis noted even greater increases, such as Pertuz with a change in AUC from 0.609 to 0.786 when using texture features (Table 5) [52].

**Table 4:** Analytical models used for breast cancer risk with breast texture features in addition to mammographic breast density for 17 studies not using the contralateral breast and reporting AUC (sorted by year)

References	Non-mammogram covariates included (e.g., age, parity, etc.)	Established confounders for density adjusted for	Prediction horizon (< 5 year/5 year/10 year)	AUC (baseline model)	Overall AUC (with texture features added)
Malkov et al. [38]	Adjusted for age, body mass index, first-degree family history, percent density, study	Age and BMI	NR	0.617	0.621
Tan et al. [43]	None	Age	NR	NR	0.730
Winkel et al. [49]	Adjusted for age	Age	NR	BIRADS density = 0.63	0.65
Eriksson et al. [29]	Percentage mammographic density, age at mammography, BMI, family history of breast cancer, HRT use	Age, BMI, HRT use, and menopausal status	2 years (for main model) and 3 years (relative risks given)	0.64	0.71 with density and interaction between percentage density and masses also included
Winkel et al. [48]	Adjusted for birth year, age at false-positive screen, invitation round at false-positive screen	Age	NR	BIRADS density = 0.65, percentage mammographic density = 0.62	0.63
Yan et al. [50]	None	None	Next sequential mammography screening	NR	0.816
Yan et al. [51]	None	None	Next sequential sequencing	NR	0.830
Gastounioti et al. [31]	Density, BMI, age	Age and BMI	NR	0.62	0.67
Heidari et al. [32]	None	Age	12 to 18 months	NR	0.68
Schmidt et al. [40]	Adjusted for age, BMI	Age and BMI	NR	Percent mammographic density = 0.620	0.662
Tagliafico et al. [42]	None	Age	NR	NR	0.567
Evans et al. [30]	None	None	3 years	NR	0.54
Hsu et al. [34]	BIRADS, density, age, race, BMI, age at first live birth, noticeable changes in breast, number of risk factors, 5-year Gail risk ≥ 1.67%	Age and BMI	NR	cv-AUC = 0.83 with BIRADS and density only	cv-AUC = 0.84
Kontos et al. [35]	Density, BMI	Age and BMI	NR for AUC model. 5-year risk from Gail and Breast Cancer Surveillance Consortium models compared by phenotype	0.80	0.84
Abdolell et al. [25]	Age, percent mammographic density	Age	Tailored estimates of current breast cancer risk	0.584	0.597
Ma et al. [36]	Age, age at menarche, menopausal status, age at first birth, parity, family history of breast cancer, breast density	Age and menopausal status	NR	0.61 for training set and 0.56 for test set	0.77 for training set and 0.75 for test set
Heine et al. [33]	Adjusted for study, age, body mass index and also with dense volume included	Age and BMI	NR	Volumetric breast density = 0.61 for first study and 0.59 for generalization study	V = 0.61, P_1_ = 0.61, p_1_ = 0.60 for first study. V = 0.59, P_1_ = 0.57, p_1_ = 0.58 for generalization study

*AUC* area under the curve, *BIRADS* Breast Imaging Reporting and Data System, *BMI* body mass index, *cv-AUC* cross-validated area under the curve, *HRT* hormone replacement therapy, *NR* not reported

**Table 5:** Analytical models used for breast cancer risk with breast texture features in addition to mammographic breast density for 5 studies using the contralateral breast (sorted by year)

References	Non-mammogram covariates included (e.g., age, parity, etc.)	Established confounders for density adjusted for	Prediction horizon (< 5 year/5 year/10 year)	AUC (baseline model)	Overall AUC (with texture features added)
Ali et al. [26]	Age, BMI, density, HRT status, parity, age at first birth	Age, BMI, HRT use, and menopausal status	NR	0.687 for apparent, 0.634 for honest	0.703 for apparent, 0.643 for honest
Wang et al. [45]	Adjusted for age, BMI	Age, BMI, HRT use, and menopausal status	NR	mC = 0.57	mC = 0.58
Perez-Benito [39]	Percent density	Age	NR	0.560	0.614
Pertuz et al. [52, 63]	None and with age, percent density	Age	NR	Density only = 0.609	0.786
Tan et al. [44]	None, with age only, and with age and BMI	Age and BMI	NR	Density = 0.52	0.68

*AUC* area under the curve, *BMI* body mass index, *HRT* hormone replacement therapy, *mC* matched concordance index, *NR* not reported

**Table 6:** Six studies of texture features and association with breast cancer risk without AUC (sorted by year)

References	Non-mammogram covariates included (e.g., age, parity, etc.)	Established confounders for density adjusted for	Prediction horizon (< 5 year/5 year/10 year)	Risk other than AUC	Statistical methods for estimating the association between features and risk
Choi et al. [28]	N/A	None	N/A	In the minimal sign group, the most common finding was normal appearing tissue (61/78), followed by benign-appearing calcification (17/78)	Chi-square test or Fisher’s exact test
Li et al. [37]	None	None	NR	OR = 1.018 (95% CI = 1.004–1.033)	t test, chi-square test, and binary logistic regression
Ward et al. [46]	None	None	NR	There was a significant correlation between a diagnosis of cancer and nodular parenchymal pattern compared to not nodular parenchymal pattern (*p* = 0.043)	Pearson’s chi-squared test with Yate’s continuity correction
Sorin et al. [41]	Adjusted for age, family history, breast density	Age	NR	The odds for breast cancer significantly increased with increased background parenchymal enhancement (OR = 2.24, 95% CI = 1.23–4.09)	Binary logistic model for generalized estimating equation
Azam et al. [27]	Adjusted for BMI, baseline mammographic density, smoking status, alcohol consumption, age at menarche, age at first birth, number of children, breastfeeding duration, oral contraceptive use, menopausal hormone therapy use, family history of breast cancer	BMI and HRT use	NR	Each additional microcalcification cluster was associated with 20% increased risk of breast cancer (HR = 1.20, 95% CI = 1.13–1.28). Women with ≥ 3 microcalcification clusters had an overall twofold increased risk of breast cancer compared to women with no clusters (HR = 2.17, 95% CI = 1.57–3.0)	Cox proportional hazard regression
Warner et al. [47]	Adjusted for age, fasting status, time of blood draw, body mass index, menopausal status, hormone therapy use, mammography read batch and also with either percent mammographic density or automated percent density	Age, BMI, HRT use, and menopausal status	NR	V was positively associated with breast cancer risk (OR = 1.15, 95% CI = 1.08–1.23 with percent mammographic density, 1.18, 95% CI = 1.07–1.31 with automated percent density)	Unconditional logistic regression

*AUC* area under the curve, *BMI* body mass index, *HR* hazard ratio, *HRT* hormone replacement therapy, *N/A* not applicable, *NR* not reported, *OR* odds ratio

Reporting on established confounders for mammographic density varied across studies, with some accounting for age, body mass index, hormone therapy, and menopausal status while others considered fewer or even none in analyses. The prediction horizon, or how far ahead a model predicts breast cancer, was only defined for 3 of the studies [29, 30, 32] and was usually the time to the next routine screening mammogram, but less than 3 years on average. Examples are summarized to give more context to the details in the tables. Eriksson et al. [29] evaluated data from the KARMA cohort that followed 70,877 women for up to 3 years after baseline mammograms. The median time from the screening mammogram to breast cancer diagnosis was 1.74 years and 433 breast cancers were diagnosed. In their analysis, they used both 2- and 3-year horizons. The AUC improved from 0.64 for the model that included age, MD, and BMI to 0.71 after adding calcifications. Heidari 2018 [32] chose 4 features associated with the asymmetry of mammogram images from a pool of 44 machine-identified features. The time horizon was 12 to 18 months, defined as the time to the next screening mammogram. A machine learning classifier was built to predict breast cancer on the subsequent mammogram, reducing the features to a vector with 4 features. The AUC improved from 0.62 to 0.68.

Not all studies reported concordance statistics for their analysis, a metric to evaluate the predictions made by an algorithm defined as the proportion of concordant pairs divided by the total number of possible evaluation pairs. We summarized six studies in Table 6 that reported association measures for texture features with breast cancer [27, 28, 37, 41, 46, 47].

Results from the assessment of the risk of bias are shown in Additional file 1: Table S1. While many studies demonstrated similar risk of bias within specific domains, there was some variability, especially for study confounding. For study confounding, studies that adjusted for age, body mass index, menopausal status, and hormone therapy were considered to have a lower risk of bias.

Study reporting impacted our ability to rate the risk of bias. None of the studies reported information to judge study attrition risk of bias, leaving all with an unclear risk of bias. Likewise, for the study population, most studies did not report on the characteristics of the source population making it difficult to determine whether the population of interest was adequately represented.

## Discussion

This review examined studies that evaluated the value of adding texture features to predict future breast cancer incidence or reported the association between these features and risk, of which we identified 28. Our findings suggest that risk prediction model performance increases when breast texture measures are added to mammographic breast density. The majority of studies evaluating future risk were limited to less than 3 years. Evidence using a longer interval from mammogram to diagnosis is necessary to be sufficient to guide breast cancer risk management or prevention [6, 53–56]. Results also demonstrate the need for a uniform approach to assess texture to enable comparison between studies.

The use of machine learning approaches to overcome variability in the assessment of BD has been reviewed recently [57]. The challenges of developing consistent approaches to the assessment of texture extend on that work. There is substantial variation in the methods used for defining and summarizing the measures of texture. No consistent approach is used to reduce the large number of predefined features, or machine-identified features, to a subset or summary for analysis. Studies also varied in their design. While most studies used a case–control design, some of these were nested in prospective cohorts, and other studies were retrospective cohort studies. Given this variation, we did not combine data across studies but note that comparison within studies shows that texture features are related to breast cancer incidence and improved concordance or AUC. Texture features are important contributors to breast cancer risk beyond mammographic breast density.

There have been some efforts on validating the texture features included in the studies in the literature. For example, the Tabár classification of parenchymal patterns has been shown to be highly reproducible [58] and several studies used a separate validation cohort. However, there is a need for more evidence evaluating these features, including external validation using different population characteristics.

There are several limitations to the studies included in this review. There was a large amount of variability in reporting established confounders of density, raising the issue of study confounding among studies that did not account for these established risk factors for density measures. Methods used may be nongeneralizable due to study specificities such as using different types of mammograms and different racial/ethnic populations. With short time periods between mammography and diagnosis of cancer, it is difficult to know whether studies using the ipsilateral breast are predicting breast cancer or merely detecting it. Some studies only provided evidence of an association rather than assessing the prediction performance of future risk, limiting their clinical translation. Large sample sizes are needed to validate associations, and a few studies had a small number of cases. Variation in the risk of bias observed in these studies reflects the variation in methods particularly in consideration of confounding. Level of reporting impacted our ability to fully assess the risk of bias in these studies.

We observed 5 studies using measures of texture that were measured or defined at the time of cancer diagnosis and from the contralateral breast [26, 39, 44, 45, 52]. Density was first evaluated as a risk factor for breast cancer using the contralateral breast at the time of diagnosis, and case–control studies have used an approach like this for decades in cancer epidemiology [59]. However, for assessing risk, examining features in the unaffected breast prior to diagnosis is a superior approach. Considering this previous work, we chose to include studies using the contralateral breast, but stratified tables in light of their limitations. We found that studies using the contralateral breast at the time of cancer diagnosis tended to report greater increases in AUC when adding parenchymal texture features.

To more effectively improve risk classification and prediction, we note the need for a harmonized strategy for assessing features across studies. The measurement challenge is a resource providing common mammogram images to identify the best method to measure mammographic density and predict breast cancer risk [60]. A similar project extends explorations to digital breast tomosynthesis and breast architecture distortions for use in screening and prediction model development [61].

In the 2016 review, Gastounioti et al. reviewed features of automated parenchymal texture analysis in relation to breast cancer risk [21]. This included details of methods to classify texture in mammographic images and its contribution to discrimination in case–control studies. Based on the review, they concluded that further research including large prospective studies is needed to establish the predictive value of parenchymal texture for ultimate inclusion in breast cancer risk prediction models. Such prediction models that extend to 5 and 10 years then require external validation to support clinical risk management. While we have a stronger base of evidence with more prospective studies in our review, our overall conclusions remain the same, though we emphasize the need for a coordinated strategy to enable greater comparisons and applicability of studies as well as studies using a longer prediction horizon.

Regarding our methods for this updated systematic review, we used the QUIPS tool [24] to assess the risk of bias. There are several limitations to the current review. The heterogeneity of the data did not allow for a meta-analysis. Additionally, systematic reviews are always subject to possible publication bias if all relevant studies have not been published. We used several strategies to reduce the risk of this including using a thorough search strategy designed by a medical librarian with expertise in searching for systematic reviews and searching clinicaltrials.gov for any ongoing studies.

For clinical use to guide precision prevention, we must identify both high-risk women for a range of risk reduction strategies [6] and low-risk women to consider the frequency of screening [62]. From this systematic literature review, we identify gaps in evidence to prioritize future studies. They include: (1) details on the prediction horizon for the risk of breast cancer; (2) other statistical approaches might also be used to assess the risk of breast cancer from the time of image acquisition that includes the texture features to the diagnosis of breast cancer such as survival analysis.

## Conclusion

The addition of parenchymal texture features to risk prediction models generally resulted in improved performance beyond mammographic density. Despite current limitations in the literature, the more widespread use of digital mammography and availability of digital images including parenchymal features offer a growing opportunity to more uniformly assess image texture features. Incorporating these features into risk prediction models can improve risk classification and risk prediction, leading to improved breast cancer risk management such as tailoring screening interval and prevention strategies to the level of risk.

## Supplementary Information

**Additional file 1.** Risk of bias assessments for 28 included studies using QUIPS (sorted by year). Complete search strategy. PRISMA checklist.

## Data Availability

Data are available upon reasonable request by contacting the corresponding author. All data relevant to the study are included in the article.
