# Supplementary material for: Studies of parenchymal texture added to mammographic breast density and risk of breast cancer: a systematic review of the methods used in the literature
Source: Breast Cancer Res. 2022 Dec 30;24:101. doi: 10.1186/s13058-022-01600-5 (PMC9805242; doi:10.1186/s13058-022-01600-5)
Supplement: Supplementary file 1 — Additional file 1. Risk of bias assessments for 28 included studies using QUIPS (sorted by year). Complete search strategy. PRISMA checklist. [file 13058_2022_1600_MOESM1_ESM.docx]

**Appendix**

Supplementary Table 1. Risk of bias assessments for 28 included studies using QUIPS (sorted by year),

| **Author** | **Year** | **Study Participation** | **Study Attrition** | **Prognostic Factor Measurement** | **Outcome Measurement** | **Study Confounding** | **Statistical Analysis and Reporting** |
| --- | --- | --- | --- | --- | --- | --- | --- |
| Choi | 2016 | Low risk of bias | Unclear risk of bias | Moderate risk of bias | Low risk of bias | High risk of bias | Moderate risk of bias |
| Malkov | 2016 | Low risk of bias | Unclear risk of bias | Moderate risk of bias | Low risk of bias | Moderate risk of bias | Low risk of bias |
| Tan | 2016 | Moderate risk of bias | Unclear risk of bias | Low risk of bias | Low risk of bias | High risk of bias | Low risk of bias |
| Winkel | 2016 | Low risk of bias | Unclear risk of bias | Moderate risk of bias | Low risk of bias | Moderate risk of bias | Low risk of bias |
| Ali | 2017 | Low risk of bias | Unclear risk of bias | Low risk of bias | Moderate risk of bias | Low risk of bias | Low risk of bias |
| Eriksson | 2017 | Low risk of bias | Unclear risk of bias | Low risk of bias | Low risk of bias | Low risk of bias | Low risk of bias |
| Wang | 2017 | Low risk of bias | Unclear risk of bias | Low risk of bias | Moderate risk of bias | Low risk of bias | Low risk of bias |
| Winkel | 2017 | Low risk of bias | Unclear risk of bias | Moderate risk of bias | Low risk of bias | Moderate risk of bias | Low risk of bias |
| Yan | 2017 (August) | Moderate risk of bias | Unclear risk of bias | Low risk of bias | Low risk of bias | High risk of bias | Low risk of bias |
| Yan | 2017 (October) | Moderate risk of bias | Unclear risk of bias | Low risk of bias | Low risk of bias | High risk of bias | Low risk of bias |
| Gastounioti | 2018 | Low risk of bias | Unclear risk of bias | Moderate risk of bias | Low risk of bias | Moderate risk of bias | Low risk of bias |
| Heidari | 2018 | Moderate risk of bias | Unclear risk of bias | Low risk of bias | Low risk of bias | Moderate risk of bias | Low risk of bias |
| Li | 2018 | Low risk of bias | Unclear risk of bias | Low risk of bias | Low risk of bias | High risk of bias | Moderate risk of bias |
| Schmidt | 2018 | Low risk of bias | Unclear risk of bias | Moderate risk of bias | Low risk of bias | Moderate risk of bias | Low risk of bias |
| Tagliafico | 2018 | Low risk of bias | Unclear risk of bias | Moderate risk of bias | Low risk of bias | Moderate risk of bias | Low risk of bias |
| Ward | 2018 | Low risk of bias | Unclear risk of bias | Moderate risk of bias | Low risk of bias | High risk of bias | Moderate risk of bias |
| Evans | 2019 | Moderate risk of bias | Unclear risk of bias | Moderate risk of bias | Low risk of bias | High risk of bias | Low risk of bias |
| Hsu | 2019 | Low risk of bias | Unclear risk of bias | Moderate risk of bias | Low risk of bias | Moderate risk of bias | Moderate risk of bias |
| Kontos | 2019 | Low risk of bias | Unclear risk of bias | Moderate risk of bias | Low risk of bias | Moderate risk of bias | Low risk of bias |
| Pérez-Benito | 2019 | Low risk of bias | Unclear risk of bias | Low risk of bias | Moderate risk of bias | Moderate risk of bias | Low risk of bias |
| Pertuz | 2019 | Low risk of bias | Unclear risk of bias | Low risk of bias | Moderate risk of bias | Moderate risk of bias | Low risk of bias |
| Tan | 2019 | Low risk of bias | Unclear risk of bias | Low risk of bias | Moderate risk of bias | Moderate risk of bias | Low risk of bias |
| Abdolell | 2020 | Low risk of bias | Unclear risk of bias | Low risk of bias | Low risk of bias | Moderate risk of bias | Low risk of bias |
| Ma | 2020 | Low risk of bias | Unclear risk of bias | Low risk of bias | Low risk of bias | Moderate risk of bias | Low risk of bias |
| Sorin | 2020 | Low risk of bias | Unclear risk of bias | Moderate risk of bias | Low risk of bias | Moderate risk of bias | Moderate risk of bias |
| Azam | 2021 | Low risk of bias | Unclear risk of bias | Low risk of bias | Low risk of bias | Moderate risk of bias | Moderate risk of bias |
| Heine | 2021 | Low risk of bias | Unclear risk of bias | Moderate risk of bias | Low risk of bias | Moderate risk of bias | Low risk of bias |
| Warner | 2021 | Low risk of bias | Unclear risk of bias | Low risk of bias | Low risk of bias | Low risk of bias | Moderate risk of bias |

QUIPS = Quality in Prognostic Studies

**Complete Search Strategies:**

**Search strategies designed and executed by Angela Hardi, MLIS**

**Embase.com**

=3,919 results on 9/9/2020 (Limited to English; editorials, letters, and notes excluded from results)

**Updated search** (date limited to 2020-present): 602 on 10/14/2021

('breast density'/exp OR ((breast NEAR/3 densit*):ti,ab,kw OR (mammary NEAR/3 densit*):ti,ab,kw OR (mammographic NEAR/3 densit*):ti,ab,kw)) AND ('mammography'/de OR mammograph*:ti,ab,kw OR mammogram*:ti,ab,kw OR mastrography:ti,ab,kw OR ‘digital breast tomosynthesis’:ti,ab,kw OR ‘x-ray breast tomosynthesis’:ti,ab,kw) NOT ('editorial'/it OR 'letter'/it OR 'note'/it) AND [english]/lim

**Ovid Medline All**

= 2694 results on 9/9/2020 (Limited to English; editorials, comments, and letters excluded)

**Updated search** (date limited to 2020-present): 440 results on 10/14/2021

(Breast Density/ OR (breast adj3 densit*).ti,ab. OR (mammary adj3 densit*).ti,ab. OR (mammographic adj3 densit*).ti,ab.) AND (Mammography/ OR mammograph*.ti,ab. OR mammogram*.ti,ab. OR mastrography.ti,ab. OR "digital breast tomosynthesis".ti,ab. OR "x-ray breast tomosynthesis".ti,ab.) NOT (comment.pt. OR editorial.pt. OR letter.pt.)

**CINAHL Plus**

=978 results on 9/9/2020; (Limited to English and these publication types: Clinical Trial, Corrected Article, Journal Article, Meta Analysis, Meta Synthesis, Practice Guidelines, Proceedings, Protocol, Randomized Controlled Trial, Research, Review, Systematic Review)

**Updated search** (dated limited to 2020-present): 135 results on 10/14/2021

((MH "Breast Tissue Density") OR AB(breast N3 densit*) OR TI(breast N3 densit*) OR AB(mammary N3 densit*) TI(mammary N3 densit*) OR AB(mammographic N3 densit*) OR TI(mammographic N3 densit*)) AND ((MH "Mammography") OR AB(mammograph*) OR TI(mammograph*) OR AB(mammogram*) OR TI(mammogram*) OR AB(mastrography) OR TI(mastrography) OR AB(“digital breast tomosynthesis”) OR TI(“digital breast tomosynthesis”) OR AB(“x-ray breast tomosynthesis”) OR TI(“x-ray breast tomosynthesis”))

**Scopus**

=3,162 results on 9/9/2020 (Limited to English; editorials, notes, letters, and book chapters excluded from results)

**Updated search** (date limited to 2020-present): 423 results on 10/14/2021

TITLE-ABS ( ( breast W/3 densit* ) OR ( mammary W/3 densit* ) OR ( mammographic W/3 densit*)) AND TITLE-ABS ( mammograph* OR mammogram* OR mastrography OR "digital breast tomosynthesis" OR "x-ray breast tomosynthesis" ) AND ( EXCLUDE ( DOCTYPE , "no" ) OR EXCLUDE ( DOCTYPE , "ch" ) OR EXCLUDE ( DOCTYPE , "le" ) OR EXCLUDE ( DOCTYPE , "ed" ) ) AND ( LIMIT-TO ( LANGUAGE , "English" ) )

**Cochrane Library**

=358 results on 9/9/2020 (1 Cochrane Protocol and 357 results from CENTRAL Trials)

**Updated Search** (date limited 2020-present in CENTRAL Trials) = 32 results on 10/14/2021

ID Search

#1 MeSH descriptor: [Breast Density] explode all trees

#2 ((breast NEAR/3 densit*) OR (mammary NEAR/3 densit*) OR (mammary NEAR/3 densit*) OR (mammographic NEAR/3 densit*)):ti,ab,kw

#3 #1 OR #2

#4 MeSH descriptor: [Mammography] explode all trees

#5 (mammograph* OR mammogram* OR mastrography OR “digital breast tomosynthesis” OR “xray breast tomosynthesis”):ti,ab,kw

#6 #4 OR #5

#7 #3 AND #6

**ClinicalTrials.gov**

= 11 results (searched the “Other terms” field) on 9/9/2020

**Updated Search** = 12 results on 10/14/2021 (1 new result, added to the Excel library)

(“breast density” OR “mammary density”) AND (mammograph* OR mammogram* OR mastrography OR “digital breast tomosynthesis” OR “x-ray breast tomosynthesis”)
